# Adaptive radiotherapy in locally advanced esophageal cancer with atelectasis: a case report

**DOI:** 10.1186/s12885-019-6505-4

**Published:** 2020-01-06

**Authors:** Katsuyuki Sakanaka, Kota Fujii, Takashi Mizowaki

**Affiliations:** Department of Radiation Oncology and Image-Applied Therapy, Graduate School of Medicine, Kyoto University, 54 Shogoin Kawahara-cho, Sakyo-ku, Kyoto, 606-8507 Japan

**Keywords:** Esophageal cancer, Mediastinal shift, Adaptive radiotherapy

## Abstract

**Background:**

To the best of our knowledge, no study has reported mediastinal shift accompanied with obstructive atelectasis due to bulky primary esophageal tumor components treated with adaptive radiotherapy and concurrent chemotherapy.

**Case presentation:**

Here we report the case of a 65-year-old male patient diagnosed with locally advanced thoracic esophageal squamous cell cancer, clinical T4bN1M0, stage IVA. Bronchoscopy and computed tomography (CT) revealed an almost complete obstruction of the lumen of the left bronchus due to compression by bulky primary esophageal tumor components. On admission, the patient presented with dyspnea and decreased arterial oxygen saturation. Chest radiography and CT on admission revealed mediastinal shift with left atelectasis, as opposed to findings from the chest radiography performed 26 days before admission. Because of the patient’s overall good condition, we recommended definitive chemoradiotherapy instead of palliative bronchial stent placement. After obtaining the patient’s consent, chemoradiotherapy was initiated on the following day and it comprised three-dimensional conformal radiotherapy with 60 Gy in 30 fractions with concurrent administration of cisplatin and 5-fluorouracil. During chemoradiotherapy, tumor location was monitored with cone-beam CT and chest radiography. Chemoradiotherapy on day 8 revealed no evidence of the mediastinal shift. CT simulation was reperformed to adjust the radiotherapy fields to account for geometrical changes induced by the absence of the mediastinal shift. Subsequently, the mediastinal shift and bronchial obstruction did not recur during the course of chemoradiotherapy. The patient completed the planned radiotherapy with concurrent and adjuvant chemotherapy, and no non-hematological grade ≥ 3 adverse events were observed. Complete response was confirmed 7 months after initiating chemoradiotherapy. Currently, no disease recurrence, dysphagia, or respiratory symptoms have been reported at 13 months after initiating chemoradiotherapy.

**Conclusions:**

In this study, a bulky primary esophageal tumor caused mediastinal shift due to ipsilateral bronchial obstruction. The close follow-up for monitoring resolution of the mediastinal shift during the course of chemoradiotherapy enabled adequate dose delivery to targets, thus reflecting the geometrical changes induced by the absence of the mediastinal shift. Adaptive radiotherapy technique was crucial for favorable patient outcomes in this challenging clinical situation.

## Background

Adaptive radiotherapy aims at adjusting the treatment plan during the course of radiotherapy to ensure correct target coverage and avoid normal tissue complications [1]. Its clinical usefulness has been reported in patients with lung cancer, atelectasis risk, pleural effusion, and obstructive pneumonitis related to lung cancer. During the course of radiotherapy, atelectasis, pleural effusion, and pneumonitis may improve or aggregate, eventually changing the geometrical location of lung tumors. Such geometrical changes in tumor location reportedly result in inaccurate dose delivery to targets and organs at risk during the course of radiotherapy for lung cancer, consequently affecting the clinical outcomes in patients [2]. Adaptive radiotherapy is necessary to adjust the radiotherapy plan when a geometrical change in tumor location occurs during the course of radiotherapy.

To the best of our knowledge, no existing studies in the literature have reported a mediastinal shift accompanied with ipsilateral obstructive atelectasis due to bulky thoracic esophageal cancer. Therefore, the usefulness of adaptive radiotherapy for treating thoracic esophageal cancer with mediastinal shift remains to be explored. The present study reports a case of locally advanced thoracic esophageal cancer with mediastinal shift accompanied with ipsilateral obstructive atelectasis due to primary esophageal tumor components successfully treated with adaptive radiotherapy plus concurrent chemotherapy.

### Case presentation

**Fig. 1:**
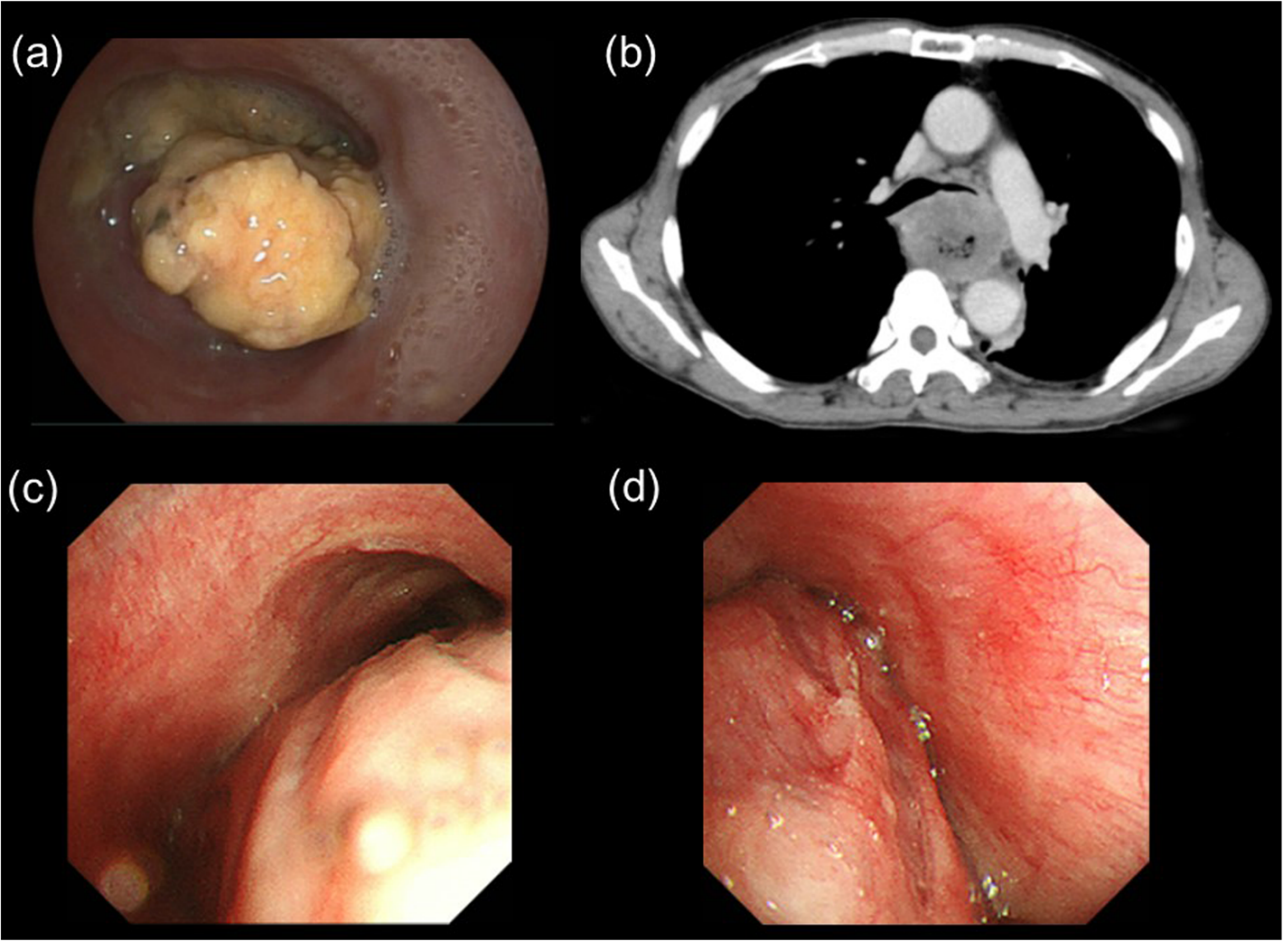
**a** Esophagogastroduodenoscopy revealed that the esophageal lumen was filled with necrotic components from the primary esophageal cancer; **b** computed tomography (CT) revealed that the left bronchus was compressed by the primary esophageal tumor components; **c** bronchoscopy revealed that the tracheal lumen at the carina level was deformed by the primary esophageal tumor components; **d** bronchoscopy revealed that the left bronchus presented with an almost complete obstruction due to compression by the primary esophageal tumor components

**Fig. 2:**
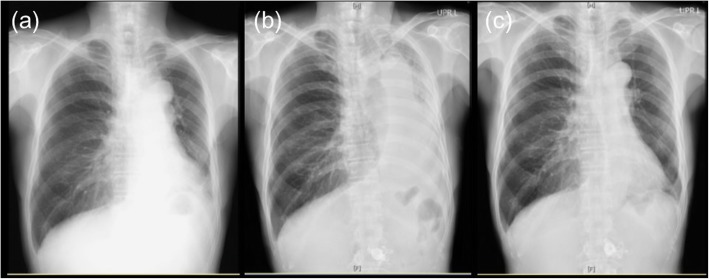
**a** Chest radiography performed 26 days before admission revealed no mediastinal shift; **b** chest radiography on the day of admission revealed a mediastinal shift and left atelectasis; **c** chest radiography performed 8 days after initiating chemoradiotherapy revealed no mediastinal shift

**Fig. 3:**
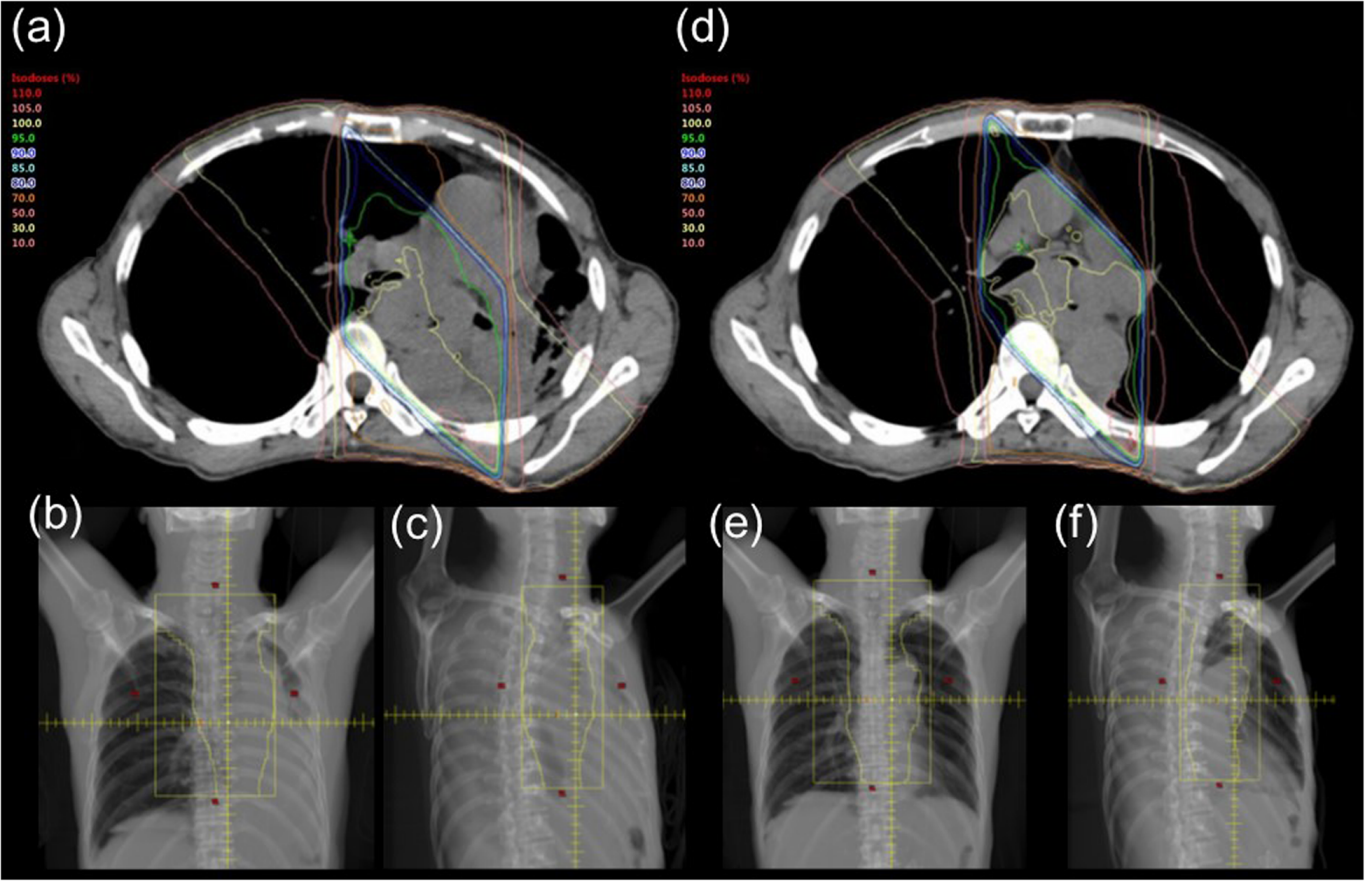
**a** Initial radiotherapy plan: axial image of dose distribution (an isodose line of 100% indicates 2 Gy per fraction); **b** initial radiotherapy plan: irradiation field of gantry angle 0°; **c** initial radiotherapy plan: irradiation field of gantry angle 315°; **d** adaptive radiotherapy plan: axial image of dose distribution showing target coverage as ensured by adaptive radiotherapy reflecting the geometrical change (iso-dose line of 100% indicates 2 Gy per fraction); **e** adaptive radiotherapy plan: irradiation field of gantry angle 0°; **f** adaptive radiotherapy plan: irradiation field of gantry angle 315°

**Fig. 4:**
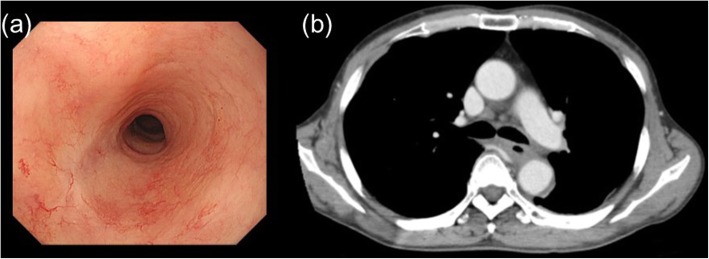
**a** Esophagogastroduodenoscopy revealed necrotic components and the disappearance of the esophageal tumor without erosion or ulceration of the esophageal mucosa at 13 months after initiating chemoradiotherapy; **b** computed tomography revealed that the airway deformation had resolved and the esophageal tumor was not observed at 13 months after initiating chemoradiotherapy

A 65-year-old male patient with a 3-month history of dysphagia was diagnosed with locally advanced thoracic esophageal squamous cell cancer, cT4bN1M0, stage IVA (Union for International Cancer Control TNM 8th edition) (Fig. 1a and b). The primary esophageal tumor components, including primary esophageal cancer and nearby metastatic lymph nodes, were bulky. The lumen of the left bronchus was almost completely obstructed by compression of the tumor masses (Fig. 1c and d). The patient was referred to our specialized hospital for treating the thoracic esophageal cancer. On the day of admission, he presented with dyspnea and decreased arterial oxygen saturation (approximately 90% under room air conditions). As opposed to the findings of chest radiography performed 26 days before admission (Fig. 2a), a mediastinal shift with left atelectasis was detected on chest radiography performed on the day of admission (Fig. 2b). Contrast-enhanced computed tomography (CT) revealed that the mediastinal shift was due to complete obstruction of the left bronchus by the primary esophageal tumor components. The patient had a good overall performance status and good organ function immediately before the left bronchial obstruction; therefore, he was recommended to undergo definitive chemoradiotherapy with curative intent rather than palliative bronchial stent placement, and the patient consented to this treatment. Chemoradiotherapy was initiated on the following day and it comprised three-dimensional conformal radiotherapy with 60 Gy in 30 fractions with concurrent administration of cisplatin (70 mg/m^2^ on day 1 and 29) and 5-fluorouracil (700 mg/m^2^ on days 1–4 and 29–32). Radiotherapy comprised four coplanar irradiation fields, including the gross tumor volumes with adequate margin and elective nodal irradiation for paraesophageal and paratracheal lymph nodes (Fig. 3a–c). Tumor location was monitored with cone-beam CT and chest radiography during the course of chemoradiotherapy. On chemoradiotherapy day 8, follow-up chest radiography revealed no evidence of the mediastinal shift (Fig. 2c). CT simulation was reperformed for adaptive radiotherapy accounting for the geometrical changes resulting from the absence of the mediastinal shift during the course of chemoradiotherapy. In this manner, the adaptive radiotherapy plan ensured correct dose delivery to the primary esophageal tumor components (Fig. 3d–f). Adaptive radiotherapy improved the target coverage, such that the doses covering 98% of the planed target volume (D98%), D50%, and D2% were 92, 100, and 105%, respectively, of the prescribed dose in the adaptive radiotherapy plan compared with 7, 97, and 106%, respectively, of the prescribed dose in the non-adaptive radiotherapy plan. After a dose delivery of 40 Gy, the irradiation fields were additionally cone downed to irradiate the gross tumors alone. After completion of the radiotherapy and concurrent chemotherapy plan, two courses of adjuvant chemotherapy were administered every 4 weeks: cisplatin (80 mg/m^2^ on day 1) and 5-fluorouracil (800 mg/m^2^ on days 1–5). The patient completed the planned treatment course with chemoradiotherapy and adjuvant chemotherapy with no grade ≥ 3 non-hematological adverse events. Complete response was confirmed by esophagogastroduodenoscopy and CT performed at 7 months after initiating chemoradiotherapy (Fig. 4a and b). At present, the patient has shown no disease recurrence, dysphagia, or respiratory symptoms at 13 months after initiating chemoradiotherapy. No radiotherapy-related adverse events were observed, except for radiation-induced grade 2 hypothyroidism.

## Discussion and conclusions

The present case report highlights two important clinical observations of our clinical case. Firstly, bronchial obstruction due to a bulky primary esophageal tumor caused a mediastinal shift accompanied with atelectasis. Secondly, adaptive radiotherapy was required to deliver correct doses to the targets, thus reflecting the geometrical changes in tumor location and organs at risk during the course of chemoradiotherapy in patients with thoracic esophageal cancer.

In the present study, bulky primary esophageal cancer components caused a mediastinal shift with bronchial obstruction and atelectasis. Bronchial obstruction, atelectasis, and pleural effusion are well-known associated findings in centrally located lung cancer [2], which have been reported in 23% of patients with locally advanced lung cancer before initiating radiotherapy by a retrospective study [2]. Contrary to the findings of lung cancer, such findings are rare in untreated esophageal cancer without distant metastases [3]. The major initial symptoms of esophageal cancer are esophagus related, such as dysphagia and weight loss [4], which help in the early diagnosis of esophageal cancer before the primary esophageal tumor components become bulky and compress the bronchi. To the best of our knowledge, the present study is the first report of this nature and it should raise awareness about the fact that left bronchial obstruction may occur due to compression by bulky esophageal primary tumors, causing a mediastinal shift accompanied with atelectasis in patients with thoracic esophageal cancer.

Chemoradiotherapy for clinical T4 esophageal cancer provides long survival [5, 6]. Successful tumor shrinkage by chemoradiotherapy for lung cancer reportedly resolves the mediastinal shift and atelectasis by reducing tumor-associated airway obstruction with a geometrical change in the tumor location [7]. The resolution rate of atelectasis after thoracic radiotherapy has been reported to be 38–90% in patients with lung cancer [8–10]. The resolution of atelectasis decreases mass shadow and density in the lung tissue with substantial changes in the normal tissue and tumor doses [11]. Similar to that observed in lung cancer with a mediastinal shift treated with radiotherapy, a geometrical change in the tumor location was observed during the course of chemoradiotherapy in the present study, thus indicating the possibility of inadequate tumor dose coverage. Inadequate dose administration reportedly affects the clinical outcomes of chemoradiotherapy for esophageal cancer [12]. In the clinical case reported in the present study, tumor location was monitored with cone-beam CT and chest radiography during the course of chemoradiotherapy, which enabled the detection of significant geometrical errors in the tumor location outside of the irradiation fields and subsequently lead to correcting these errors through an adaptive radiotherapy plan to deliver an adequate dose to the targets. The present case study demonstrated that chemoradiotherapy with a curative intent is effective for treating locally advanced unresectable esophageal cancer, but this study is unique because it also shows that adaptive radiotherapy warrants an accurate dose delivery to targets in such cases.

In conclusion, a bulky primary esophageal tumor caused mediastinal shift with ipsilateral bronchial obstruction in the present clinical case. A close follow-up with cone-beam CT or chest radiography during the course of chemoradiotherapy to confirm resolution of the mediastinal shift was key in ensuring the correct dose delivery to targets. Overall, chemoradiotherapy with adaptive radiotherapy appears to yield a curative treatment strategy for patients in such difficult clinical situations.

## Data Availability

The datasets used during the current study are available on request from the corresponding author.

## Abbreviation

CT: Computed tomography
